# Supplementary material for: Senescent cells suppress macrophage-mediated corpse removal via upregulation of the CD47-QPCT/L axis
Source: J Cell Biol. 2022 Dec 2;222(2):e202207097. doi: 10.1083/jcb.202207097 (PMC9723804; doi:10.1083/jcb.202207097)
Supplement: Table S1 — shows lists all materials (e.g. chemicals, antibodies, primers). [file JCB_202207097_TableS1.docx]

**Table S1**

| **REAGENT or RESOURCE** | **SOURCE IDENTIFIER** | **SOURCE** |
| --- | --- | --- |
| Aliphatic Amine latex, 2% w/v 1µM | A37362 | Invitrogen |
| anti-CD24 FAB | TAB-007LC-F(E) | Creative BioLabs |
| anti-CD47 FAB | TAB-274LC-F(E) | Creative BioLabs |
| anti-SIRPa FAB | PABJ-0078-F(E) | Creative BioLabs |
| BSA | A3059-50G | Sigma |
| CellTracker™ Green CMFDA Dye | C2925 | Invitrogen |
| CypHer5E NHS Ester | PA15401 | Cytiva |
| Cytolight Rapid dye | 4705 | Sartorius |
| D-(+)-Glucose Solution (45%) | G8769-100ML | Sigma |
| DMEM + Glucose +Pyruvat | 61966-021 | Gibco |
| DMEM + Glucose -Pyruvat | 61965-026 | Gibco |
| DNAase | DN25-10MG | Thermo Fisher |
| Etoposide | E1383 | Sigma |
| FBS, Qualified, HI (origine: Brazil) | 10500-064 | Gibco |
| Fc Block | 31880 | Thermo Fisher |
| Fms-like tyrosine kinase 3 ligand (FLT3L) | 250‑31L | Peprotech |
| HEPES | 15630-080 | Gibco |
| Hoechst | H3570 | Invitrogen |
| IL13 (human) | 213-ILB-025/CF | R&D |
| IL13 (mouse) | 413-ML/CF | R&D |
| IL4 (human) | 204-IL-010/CF | R&D |
| IL4 (mouse) | 404-ML-010/CF | R&D |
| IL5 (mouse) | 215-15 | Peprotech |
| IncuCyte pHrodo Red cell Labeling Kit | 4649 | Sartorius |
| L-glutamine | 25030081 | Gibco |
| LPS (E.coli) | L2880 | Sigma |
| LPS (Salmonella) | L6143-1MG | Sigma |
| M-CSF | 130-096-491 | Miltenyi |
| Milk powder | 42590,01 | SERVA |
| N-2-hydroxyethylpiperazine-N′-2-ethanesulfonic acid | L1613 | Biochrome |
| Non-essential aminoacids | 11140-035 | Gibco |
| NSC-88777 | 565851-10MG | Merck |
| Palbociclib | PZ0383 | Sigma |
| Penicillin/Streptomycin | 15070-063 | Gibco |
| pHodo^TM^ E.coli Bioparticles^TM^ conjugate for phagocytosis | P35361 | Invitrogen |
| PKH26 Linker-Kit | PKH26GL-1KT | Sigma |
| Ponceau S Solution | P7170-1L | Sigma |
| RPMI 1640 Medium | 61870-010 | Gibco |
| sodium pyruvate | 11360070 | Gibco |
| stem cell factor (SCF) | 250‑03 | Peprotech |
| TBS | T5912-1L | Sigma-Aldrich |
| Towbin 10x | 42558,02 | SERVA |
| Trypsin 0.25% | 25200056 | Gibco |
| Tween 20 | P1379-100ML | Sigma |
| Western Lightning Plus ECL | NEL104001EA | Perkin Elmer |
| β‑mercaptoethanol | 31350-010 | Thermo Fisher |

| **ANTIBODIES** | **SOURCE IDENTIFIER** | **SOURCE** | **SPECIES** | **DILUTION** |
| --- | --- | --- | --- | --- |
| Alexa Fluor® 594 anti-mouse CD24 Antibody | 101834 | Biolegend | Rat | 5µg/ml |
| Alexa Fluor® 647  anti-mouse CD22  Antibody | 126107 | Biolegend | Rat | 10µg/ml |
| Anti-human CD47 | AF4670 | R&D | Sheep | 1:200 |
| Anti-human SHP-1 | MA5-11669 | Invitrogen | Mouse | 1:100 |
| Anti-mouse Alexa 488 | A32723 | Invitrogen | Goat | 1:500 |
| Anti-mouse CD47 | 127507 | Biolegend | Rat | 1:100 |
| Anti-mouse CD47 | AF1866 | R&D | Goat | 1:500 |
| Anti-mouse CD47 (IF) | PA5-116827 | Thermo Fisher | Rabbit | 1:100 |
| Anti-mouse F4/80 | 123115 | Biolegend | Rat | 1:100 |
| Anti-mouse SHP-1 | PA5-27803 | Thermo Fisher | Rabbit | 1:200 |
| Anti-Rabbit Alexa Fluor 488 | A-11008 | Thermo Fisher | Goat | 1:1000 |
| Anti-rabbit HRP | RPN 4301 | Amersham | Goat | 1:2500 |
| Anti-rat Alexa 568 | A11077 | Invitrogen | Goat | 1:500 |
| Anti-rat Alexa 647 | A21247 | Invitrogen | Goat | 1:500 |
| anti-sheep Alexa Fluor 488 | A11015 | Invitrogen | Donkey | 1:500 |
| Anti-sheep HRP | A3415-1ML | Sigma | Donkey | 1:5000 |
| CD22 | ab218340 | Abcam | Mouse | 1:250 |
| CD24 | ab179821 | Abcam | Rabbit | 1:1000 |
| CD24 | MA5-11828 | Invitrogen | Mouse | 1:30 |
| CD45 | MA5-17687 | Invitrogen | Rat | 1:500 |
| GAPDH (14C10) | 2118 | CST | Rabbit | 1:2000 |
| Hoechst 33342 | H3570 | Invitrogen |  | 1:500 |
| PE anti-mouse CD170 (Siglec-F) Antibody | 155505 | Biolegend | Rat | 1:100 |
| PE anti-mouse CD47 antibody | 127507 | BioLegend | Rat | 1:100 |
| Phalloidin | 8878 | Cell Signaling |  | 1:20 |

| **TaqMan GENEXPRESSION ASSAYS** | **SOURCE IDENTIFIER** | **SOURCE** |
| --- | --- | --- |
| Taqman Primers: CDKN1A | 4351370 / Hs00355782_m1 | Thermo Fisher |
| Taqman Primers: IL6 | 4351370 / Hs00236937_m1 | Thermo Fisher |
| Taqman Primers: CXCL1 | 4351370 / Hs00236937_m1 | Thermo Fisher |
| Taqman Primers: MKI67 | 4351370 / Hs00606991_m1 | Thermo Fisher |
| Taqman Primers: CD47 | 4351370 / Hs00179953_m1 | Thermo Fisher |
| Taqman Primers: QPCT | 4351370 / Hs00202680_m1 | Thermo Fisher |
| Taqman Primers: QPCTL | 4351370 / Hs01012164_gH | Thermo Fisher |
| Taqman Primers: Cdkn1a | 4351370 / Mm00432448_m1 | Thermo Fisher |
| Taqman Primers: Il6 | 4351370 / Mm00446190_m1 | Thermo Fisher |
| Taqman Primers: Cxcl1 | 4351370 / Mm04207460_m1 | Thermo Fisher |
| Taqman Primers: Mki67 | 4351370 / Mm01278617_m1 | Thermo Fisher |
| Taqman Primers: HPRT | 4448490 / Hs02800695_m1 | Thermo Fisher |
| Taqman Primers: Hprt | 4448490/ Mm03024075_m1 | Thermo Fisher |
| Taqman Primers: Cd47 | 4351370 / Mm00495011_m1 | Thermo Fisher |
| Taqman Primers: Qpct | 4351370 / Mm01225793_m1 | Thermo Fisher |
| Taqman Primers: Qpctl | 4351370 / Mm00482770_m1 | Thermo Fisher |

| **CELL LINES** | **SOURCE IDENTIFIER** | **SOURCE** |
| --- | --- | --- |
| Jurkat | TIB-152 | ATCC |
| Raji | CCL-86 | ATCC |
| Panc1 | CRL-1469 | ATCC |
| A549 | CCL-185 | ATCC |
| 3T3 | CRL-1658 | ATCC |
| TK-1 | CRL-2396 | ATCC |

| **BIOLOGIGAL SAMPLES** | **SOURCE IDENTIFIER** | **SOURCE** |
| --- | --- | --- |
| blood from healthy human donors |  | Boehringer Ingelheim |
| DHLF-IPF - Diseased Human Lung Fibroblasts, Idiopathic Pulmonary Fibrosis | CC-7231 | Lonza |
| LX-2 Hepatic Stellate Cell | SCC064 | Sigma-Aldrich |
| NHLF - Human Lung Fibroblasts | CC-2512 | Lonza |
| SAEC – Human Small Airway Epithelial Cells | CC-2547 | Lonza |

| **CRITICAL COMMERTIAL KITS** | **SOURCE IDENTIFIER** | **SOURCE** |
| --- | --- | --- |
| BrdU Assay | 11647229001 | Roche |
| CXCL1 ELISA | DY275 | R&D Systems |
| High Capacity Kit cDNA | 4368813 | Thermo |
| MagMAX™-96 Total RNA Isolation Kit | AM1830 | Thermo Fisher |
| MSD customised | K15067L-2, K151AEL-2 | Mesoscale Diagnostics |
| PathHunter® Bioassay Detection Kit | 93-0933 | Eurofins |
| Senescence ß Galactosidase Staining Kit | 9860S | Cell Signaling |
| Senso Light (QPCT Enzyme assay) | 72230 | AnaSpec |
| TaqManTM Fast Advanced Master Mix | 4444557 | Thermo Fisher |

| **SOFTWARE AND ALGORITHMS** | **SOURCE IDENTIFIER** | **SOURCE** |
| --- | --- | --- |
| Prism | Version 9 | GraphPad |
| Adobe Illustrator | CC 2021 | Adobe |
| QuantStudioTM Real-Time PCR Software | Version 1.3 | Thermo Fisher |
| ZEN blue edition | Version 3.1 | Zeiss |
| ZEN black edition |  | Zeiss |
| Axio Vision SE64 | Version 4.9 | Zeiss |
| IncuCyte 2021B | Version 2021B | Sartorius |
| XStella Camera Control | Version 2.1.8.415 | Reytest Isotope Messgeraete GmbH |
| AIDA Imaging analyser | Version 4.22 | Reytest Isotope Messgeraete GmbH |
| FlowJo | Version 10 | BD |
