# Supplementary material for: Senescent cells suppress macrophage-mediated corpse removal via upregulation of the CD47-QPCT/L axis
Source: J Cell Biol. 2022 Dec 2;222(2):e202207097. doi: 10.1083/jcb.202207097 (PMC9723804; doi:10.1083/jcb.202207097)

Source Data F7C

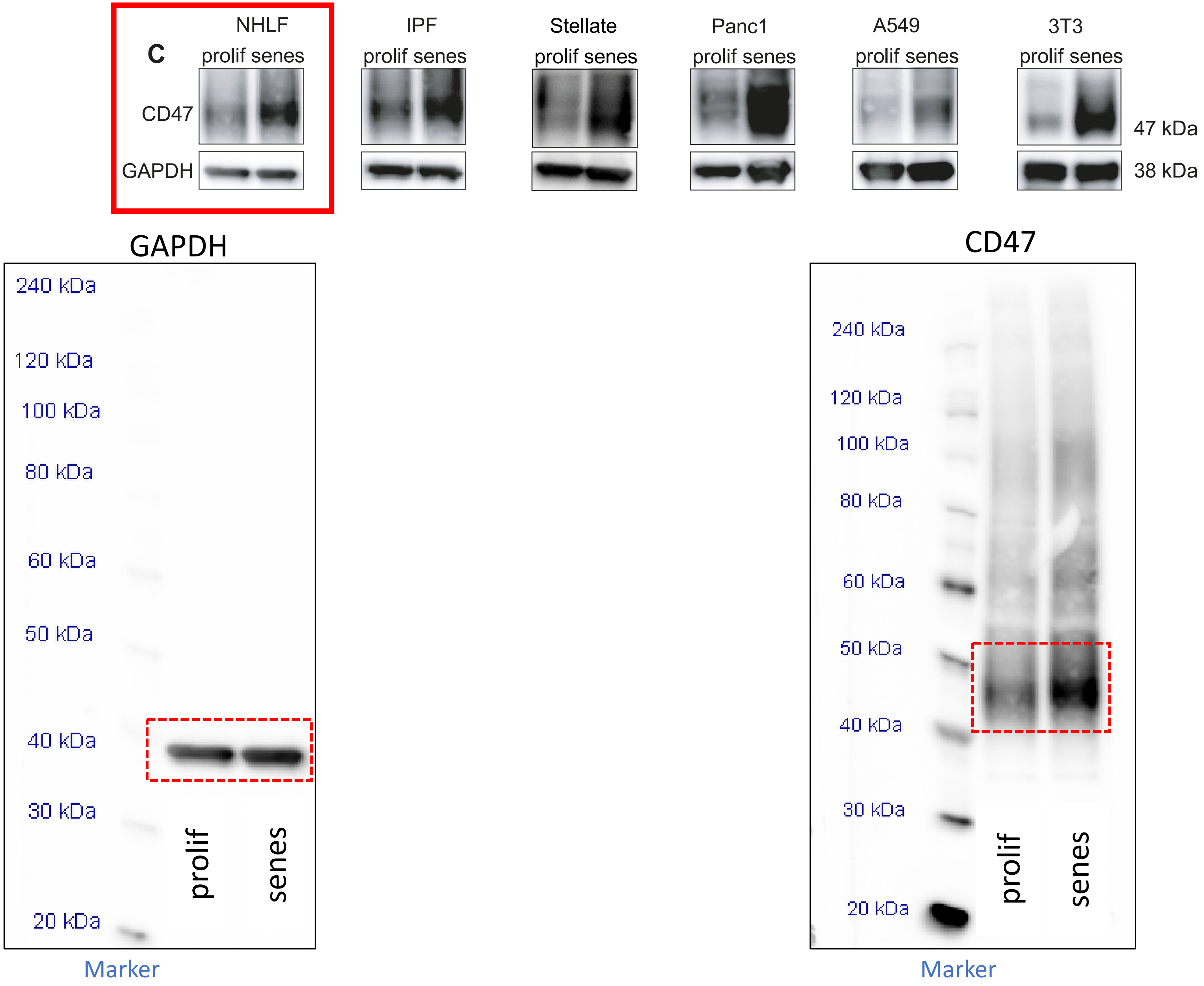

Source Data F7C

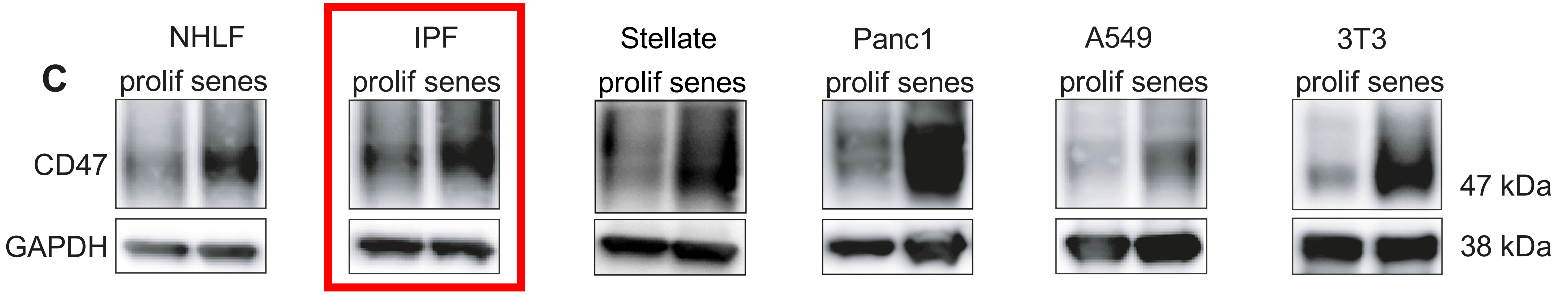

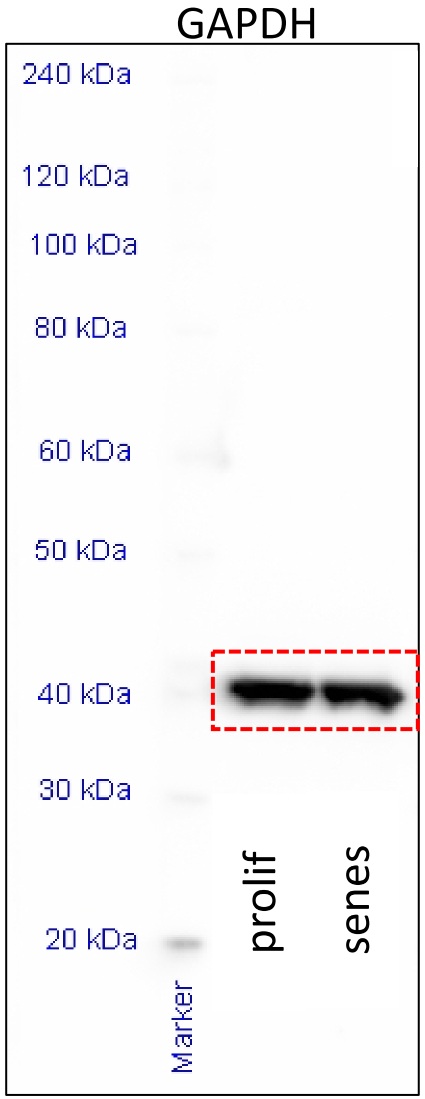

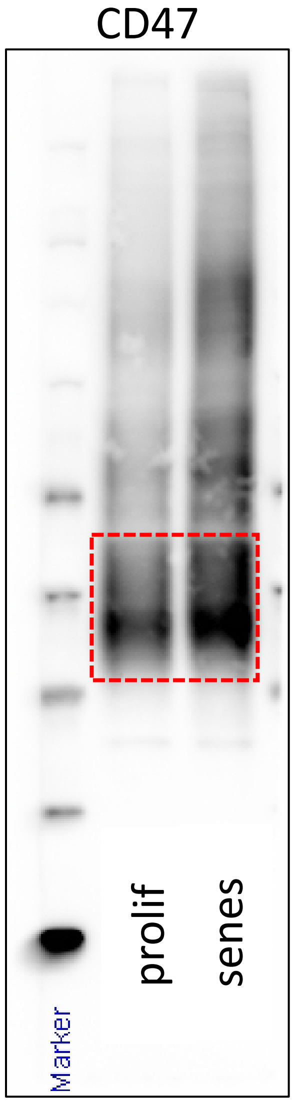

Source Data F7C

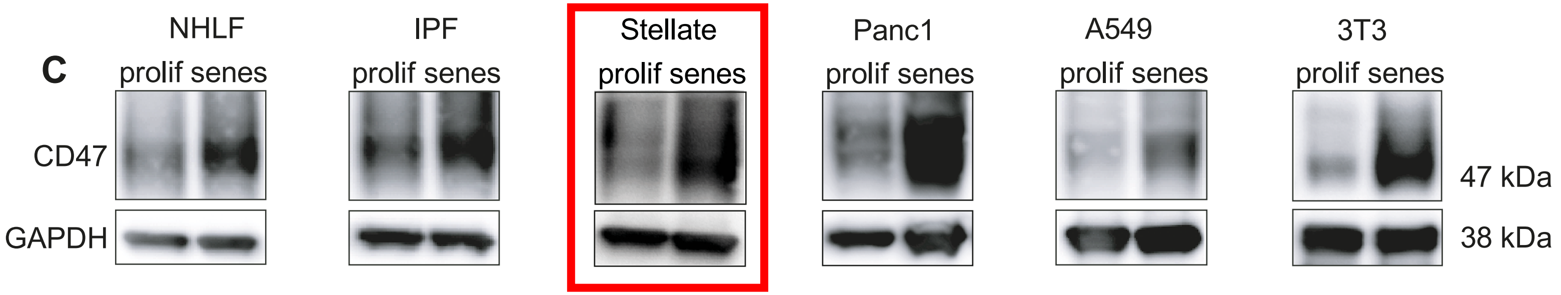

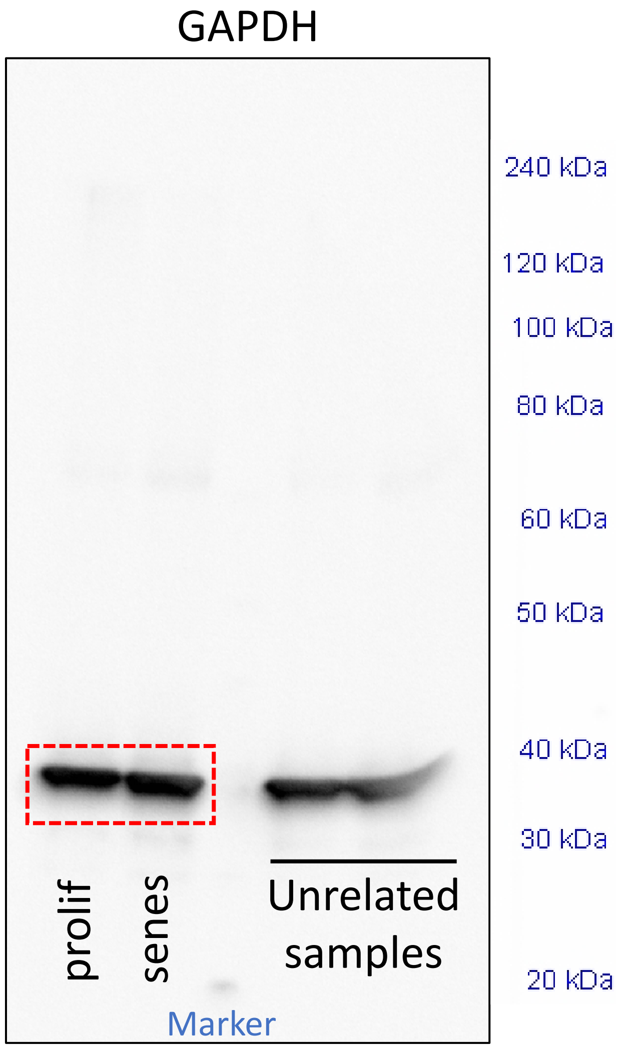

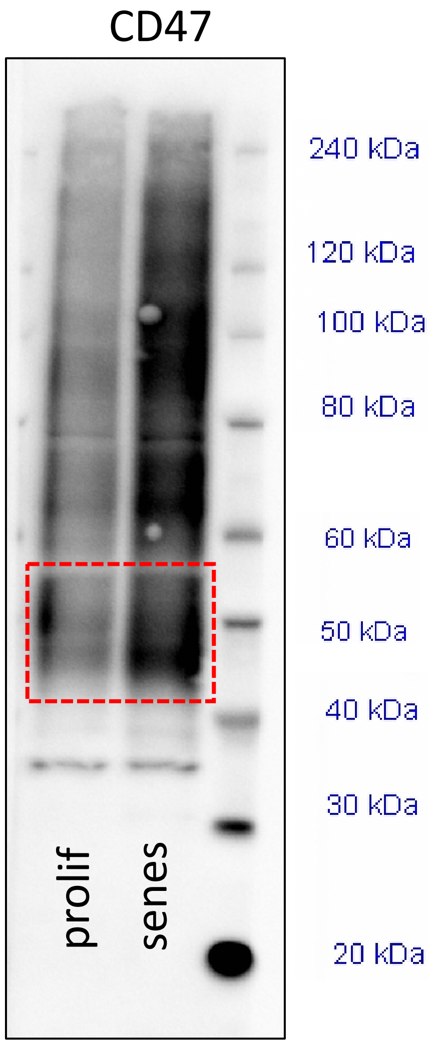

Source Data F7C

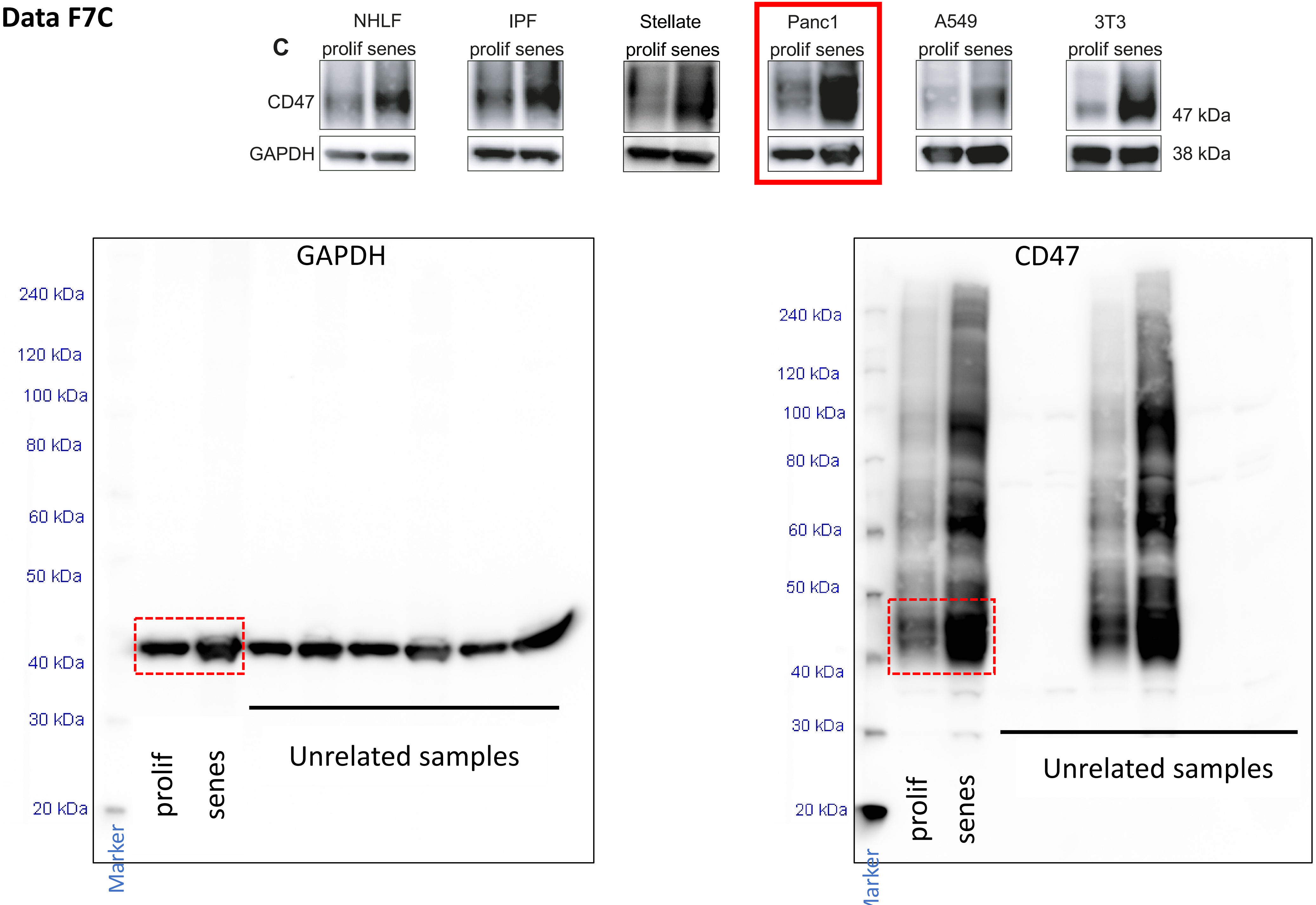

Source Data F7C

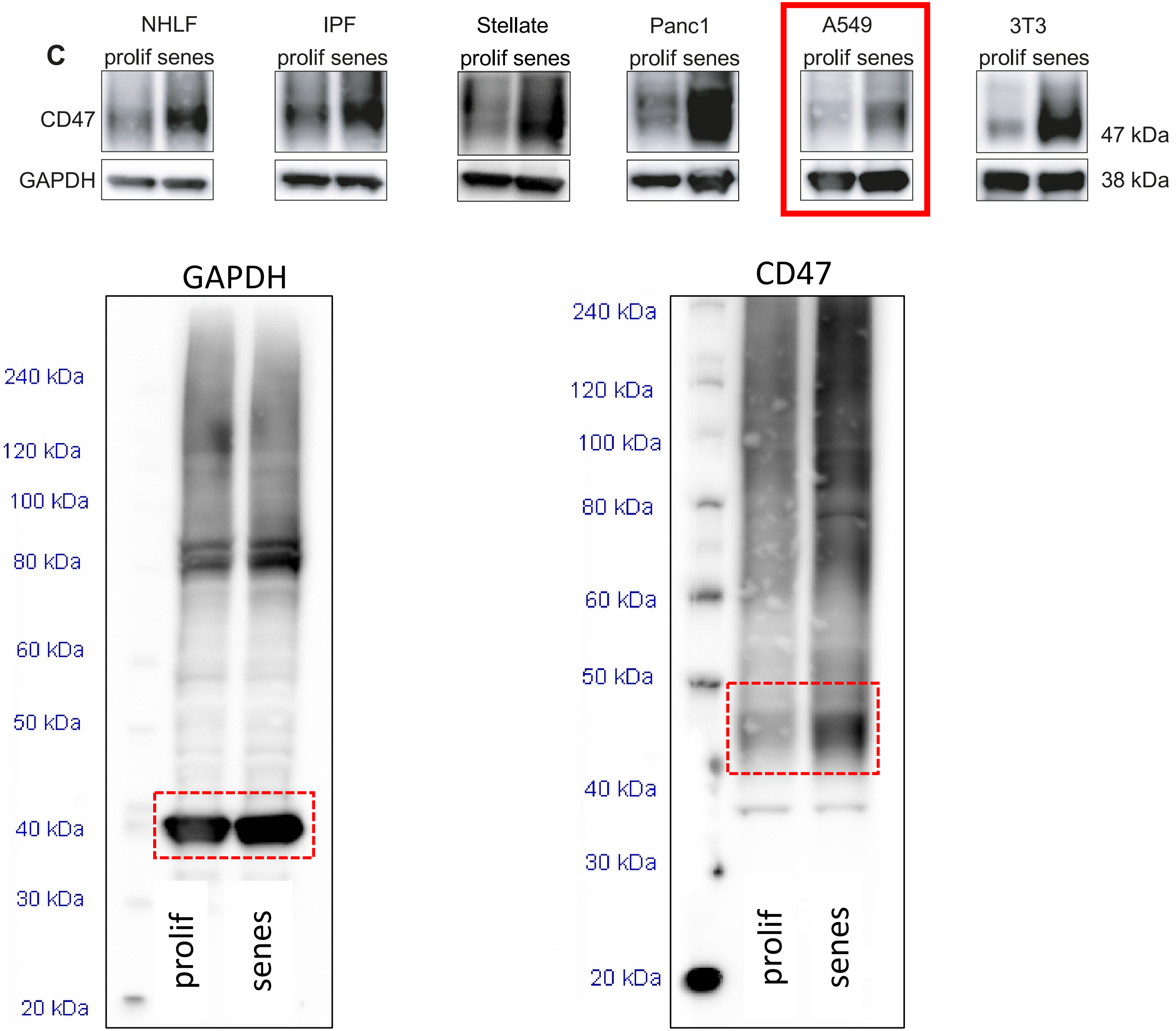

Source Data F7C

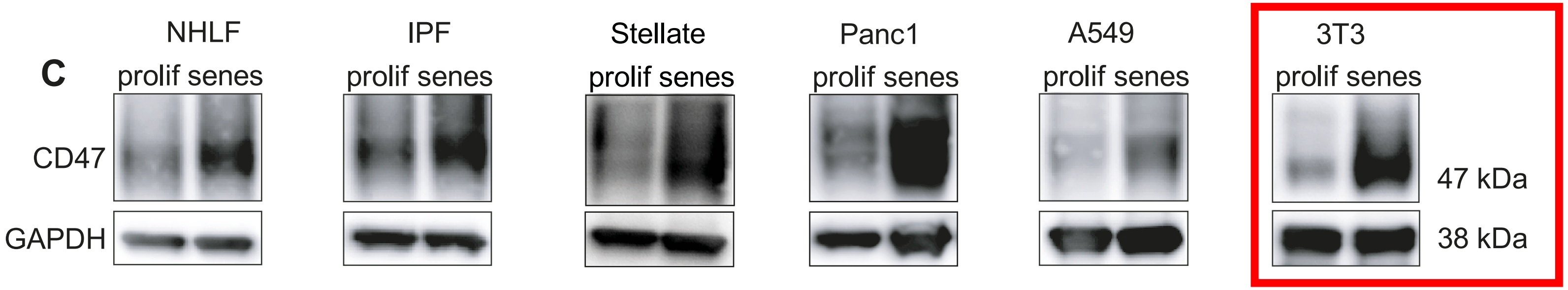

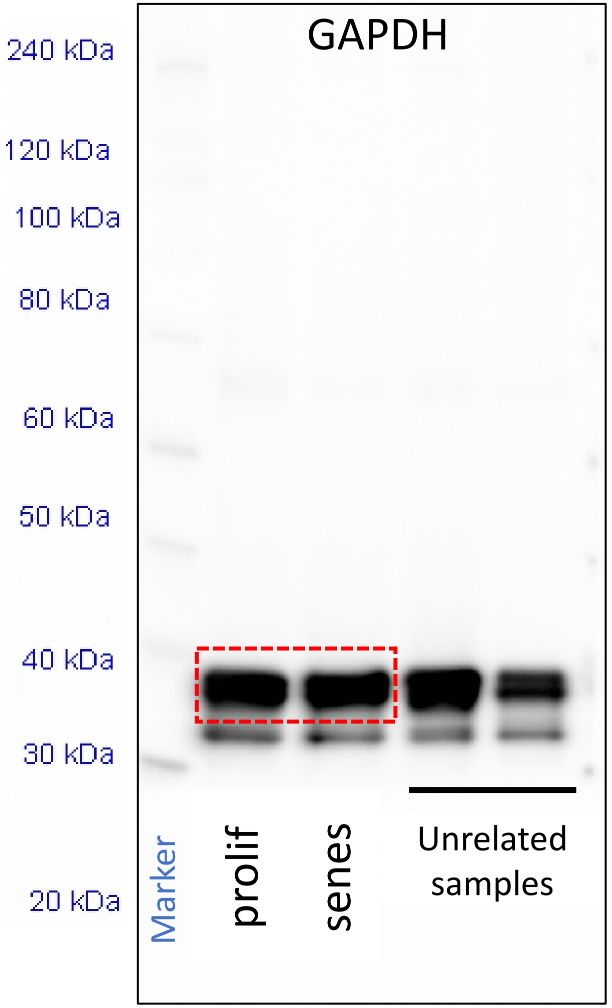

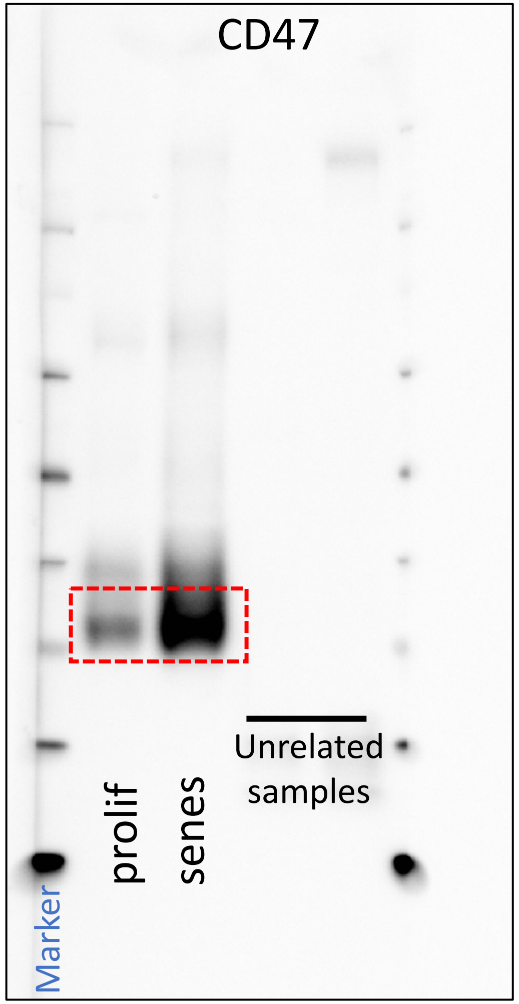

Source Data F7E

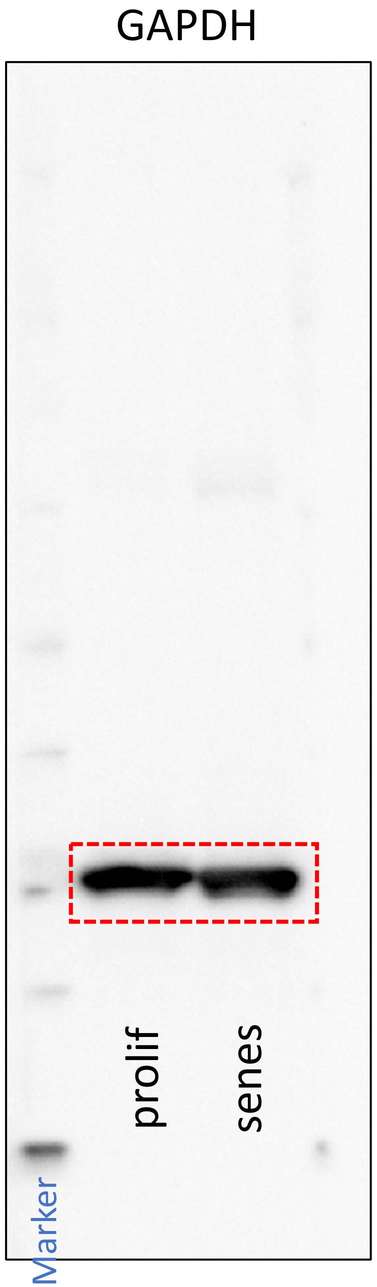

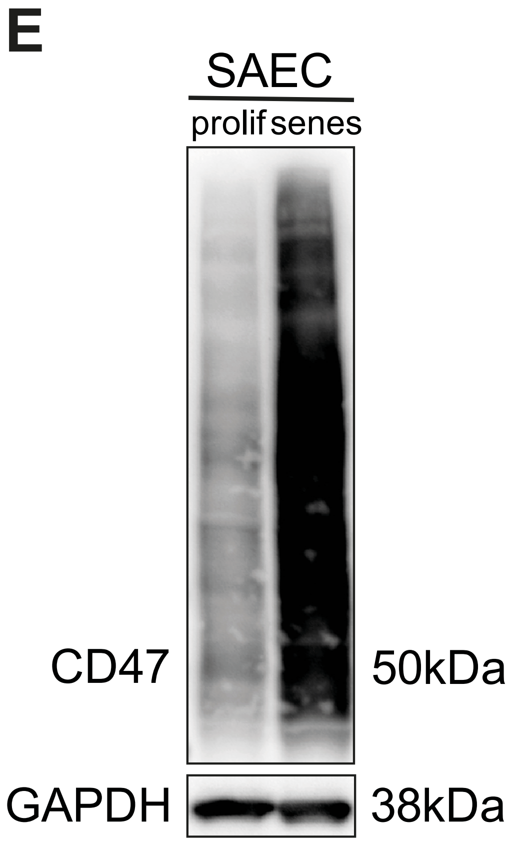

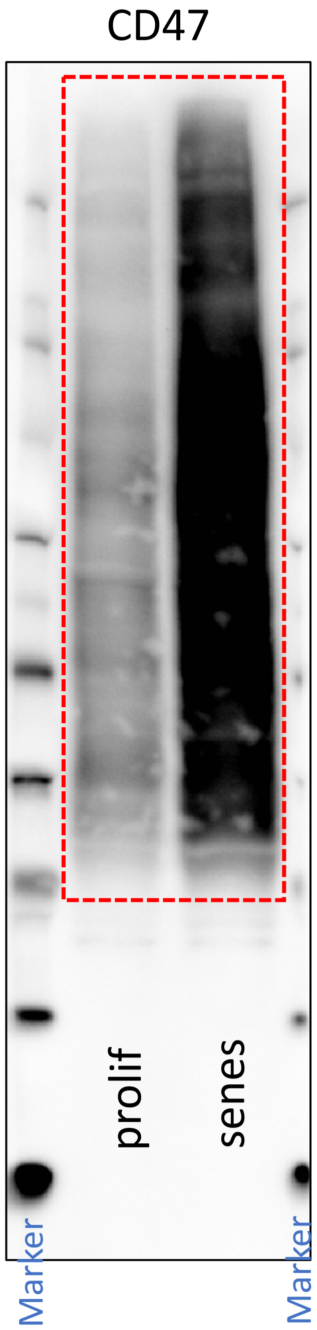

Source Data F7G

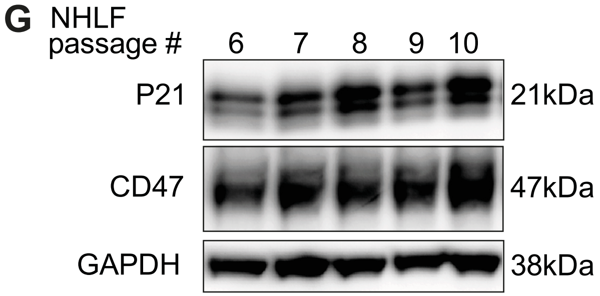

Membrane cutted at ~25kDa:
upper part → **GAPDH**

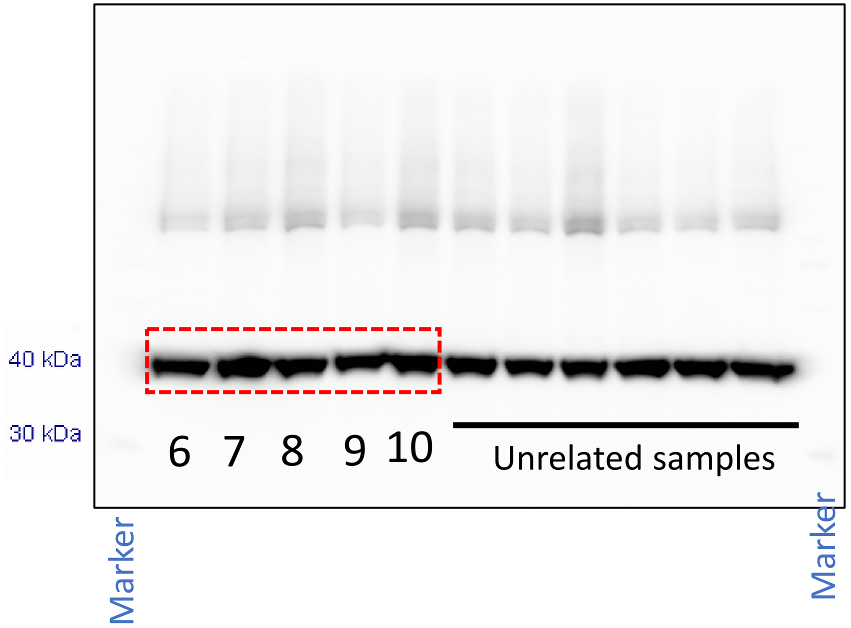

Lower part → **P21**

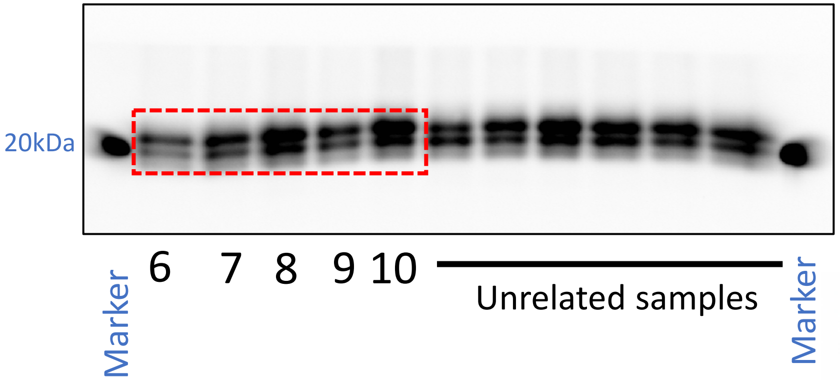

CD47

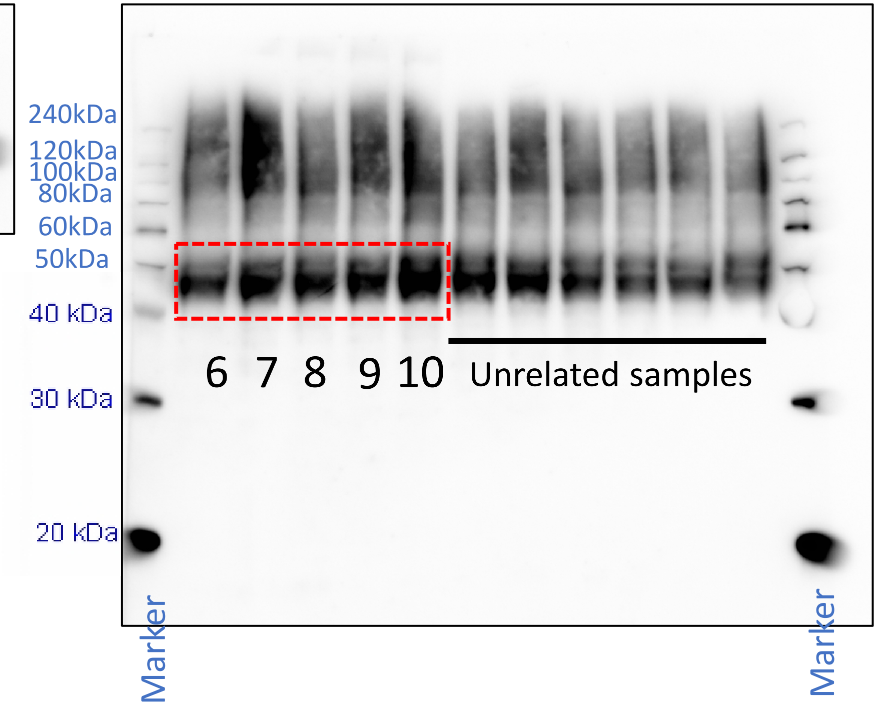

Source Data F7G

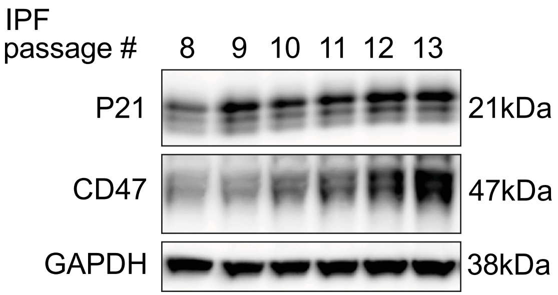

Membrane cutted at ~25kDa:
upper part → **GAPDH**

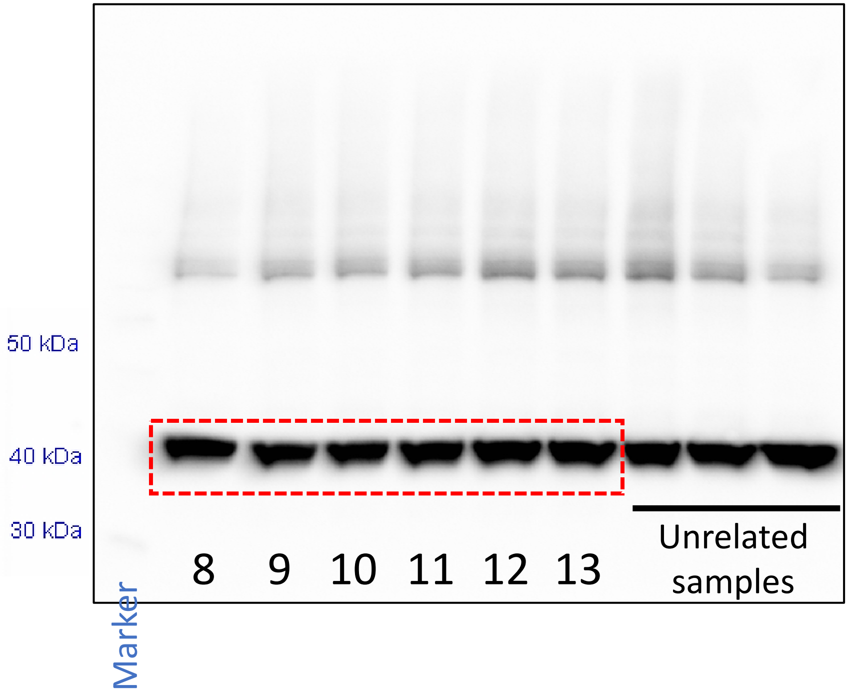

Lower part → **P21**

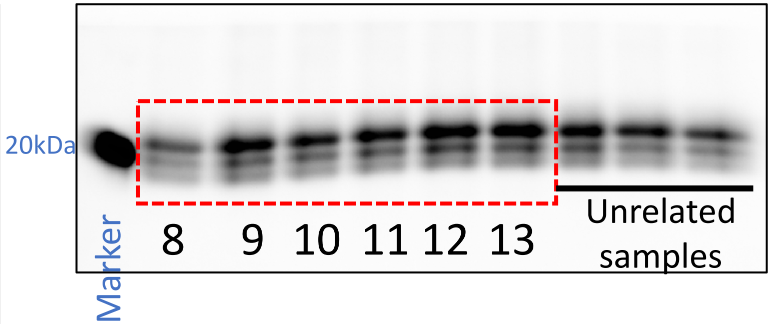

CD47

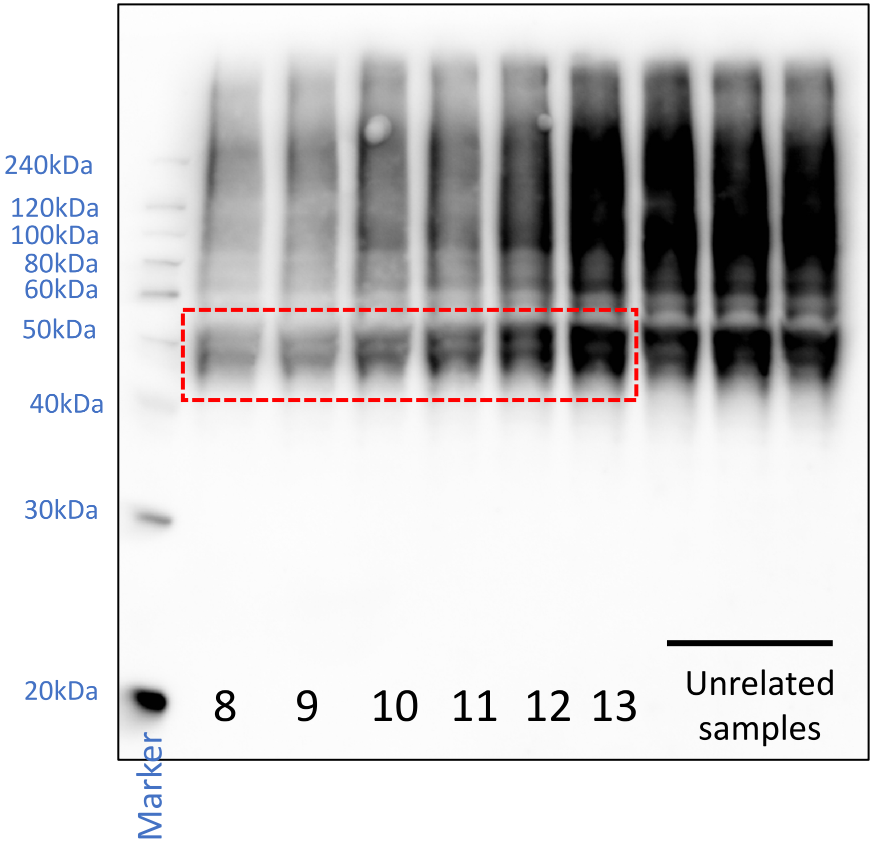

Supplement: SourceData F7 — is the source file for Fig. 7. [file JCB_202207097_SourceDataF7.pdf]
