# Supplementary figures and images for: Senescent cells suppress macrophage-mediated corpse removal via upregulation of the CD47-QPCT/L axis
Source: J Cell Biol. 2022 Dec 2;222(2):e202207097. doi: 10.1083/jcb.202207097 (PMC9723804; doi:10.1083/jcb.202207097)

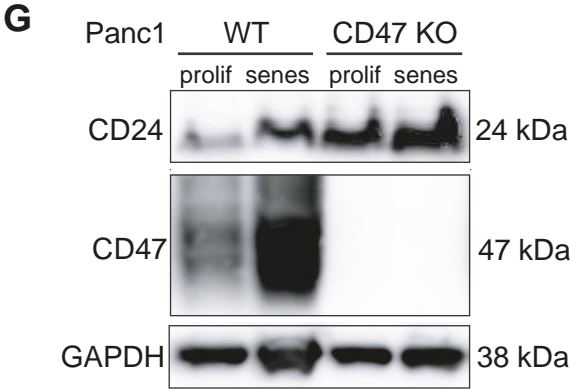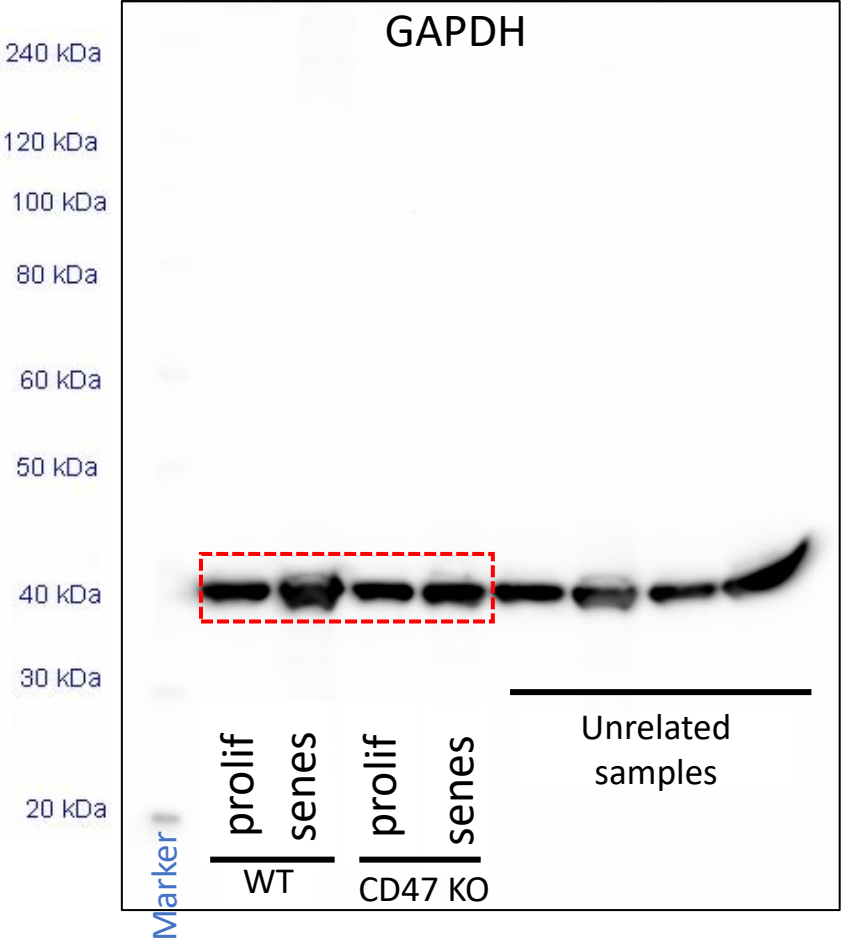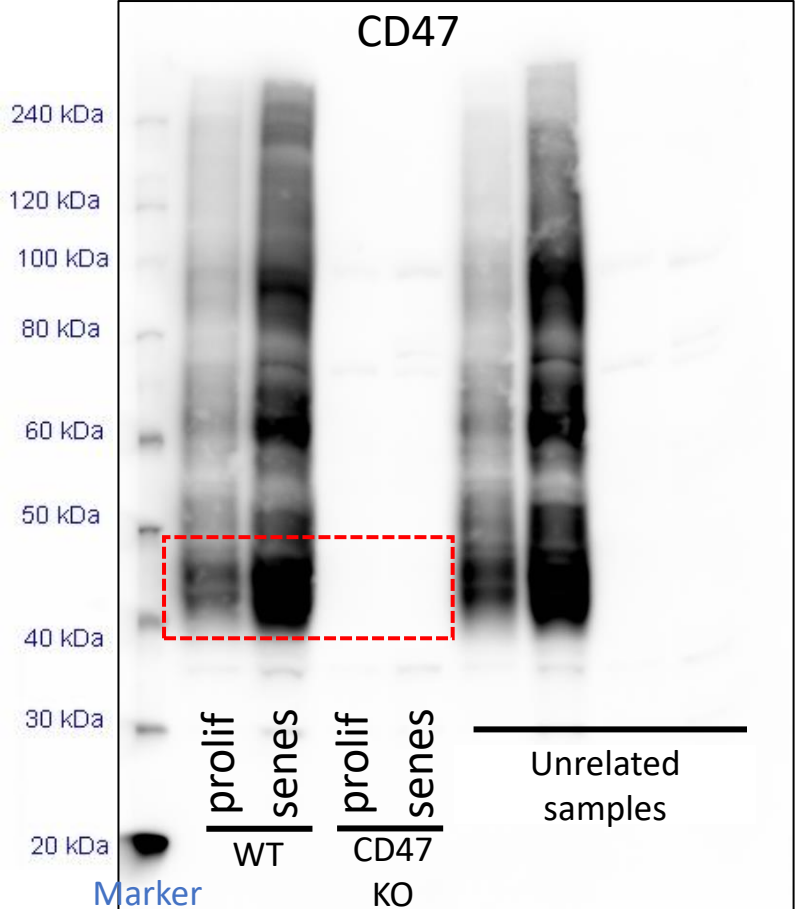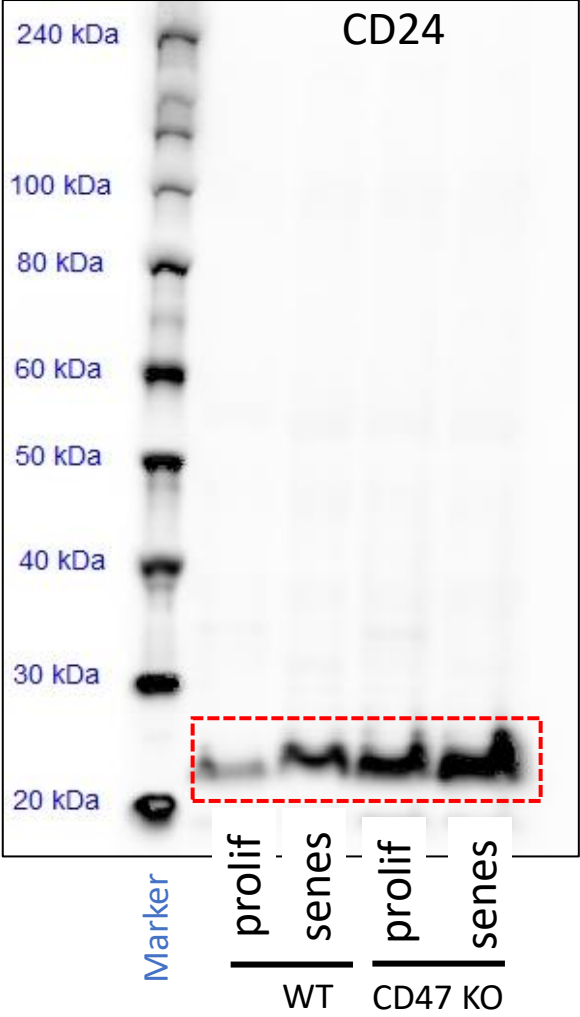

Supplement: SourceData F8 — is the source file for Fig. 8. [file JCB_202207097_SourceDataF8.pdf]
