# Supplementary material for: Senescent cells suppress macrophage-mediated corpse removal via upregulation of the CD47-QPCT/L axis
Source: J Cell Biol. 2022 Dec 2;222(2):e202207097. doi: 10.1083/jcb.202207097 (PMC9723804; doi:10.1083/jcb.202207097)

Source Data F7E

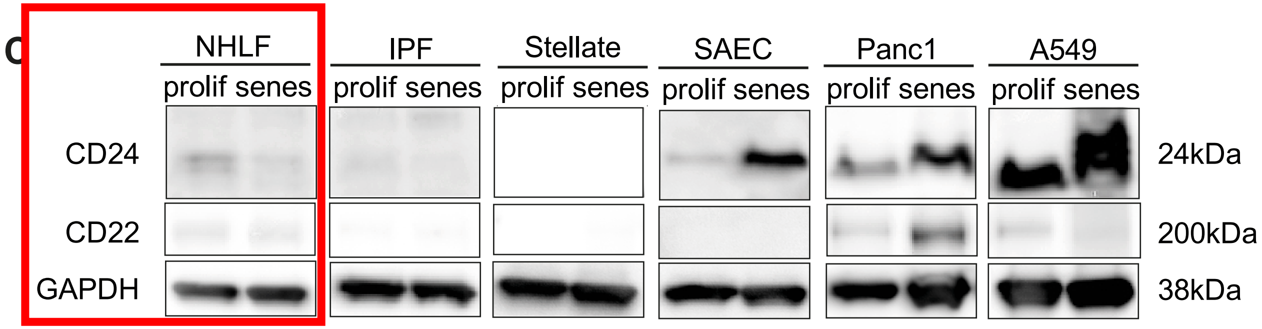

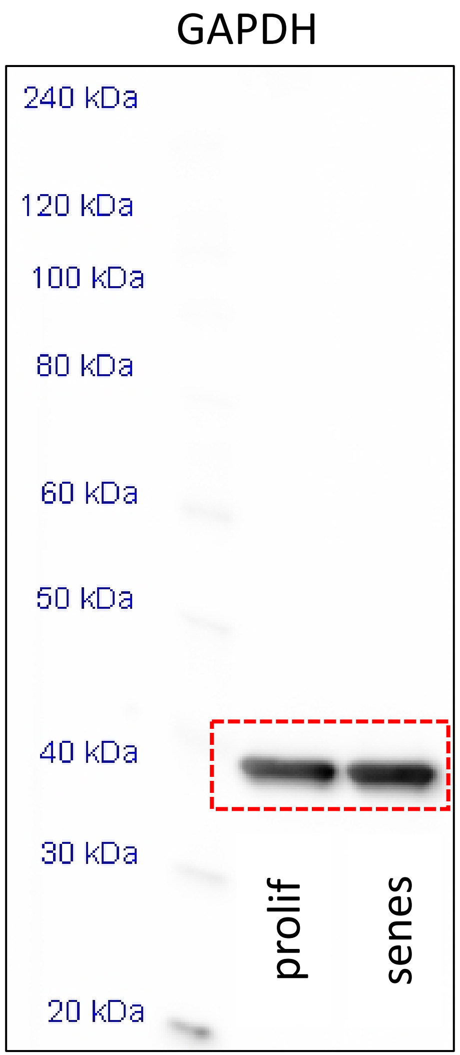

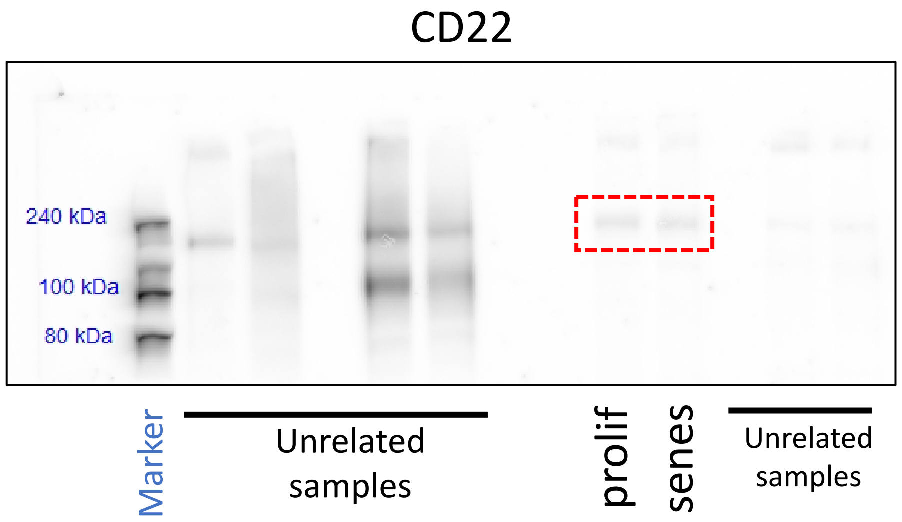

* Membrane cut at ~75kDa; lower part incubated with unrelated antibody

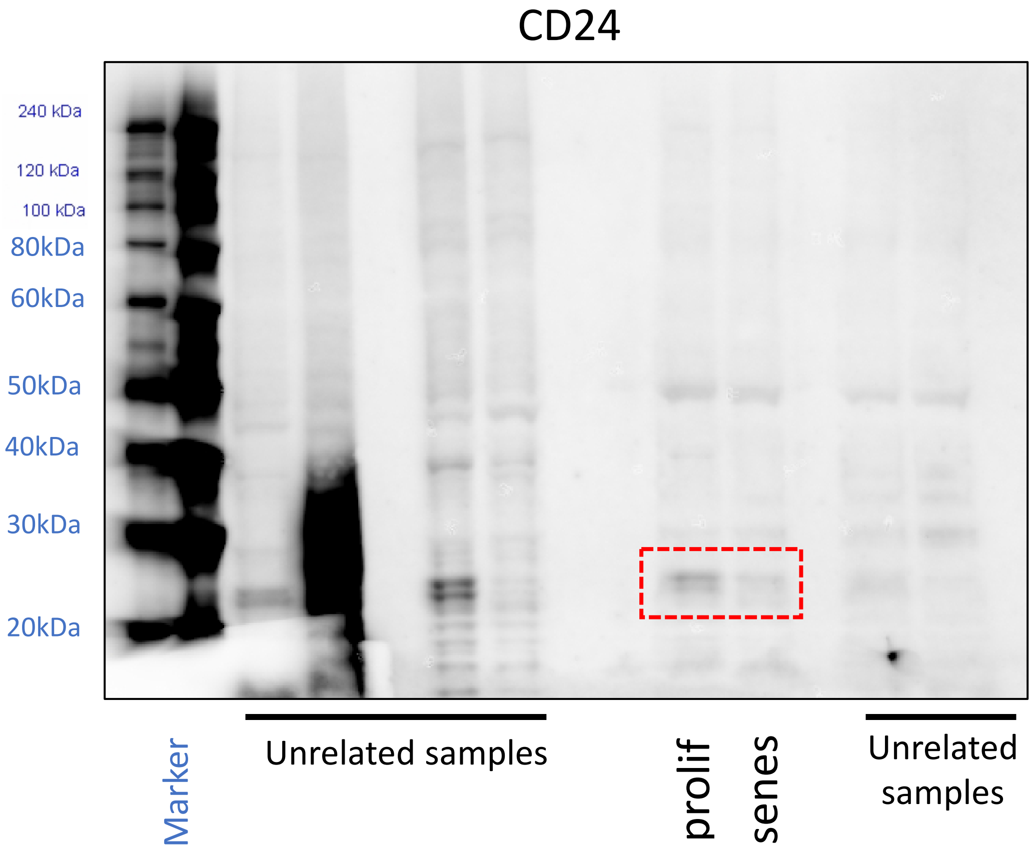

Source Data F7E

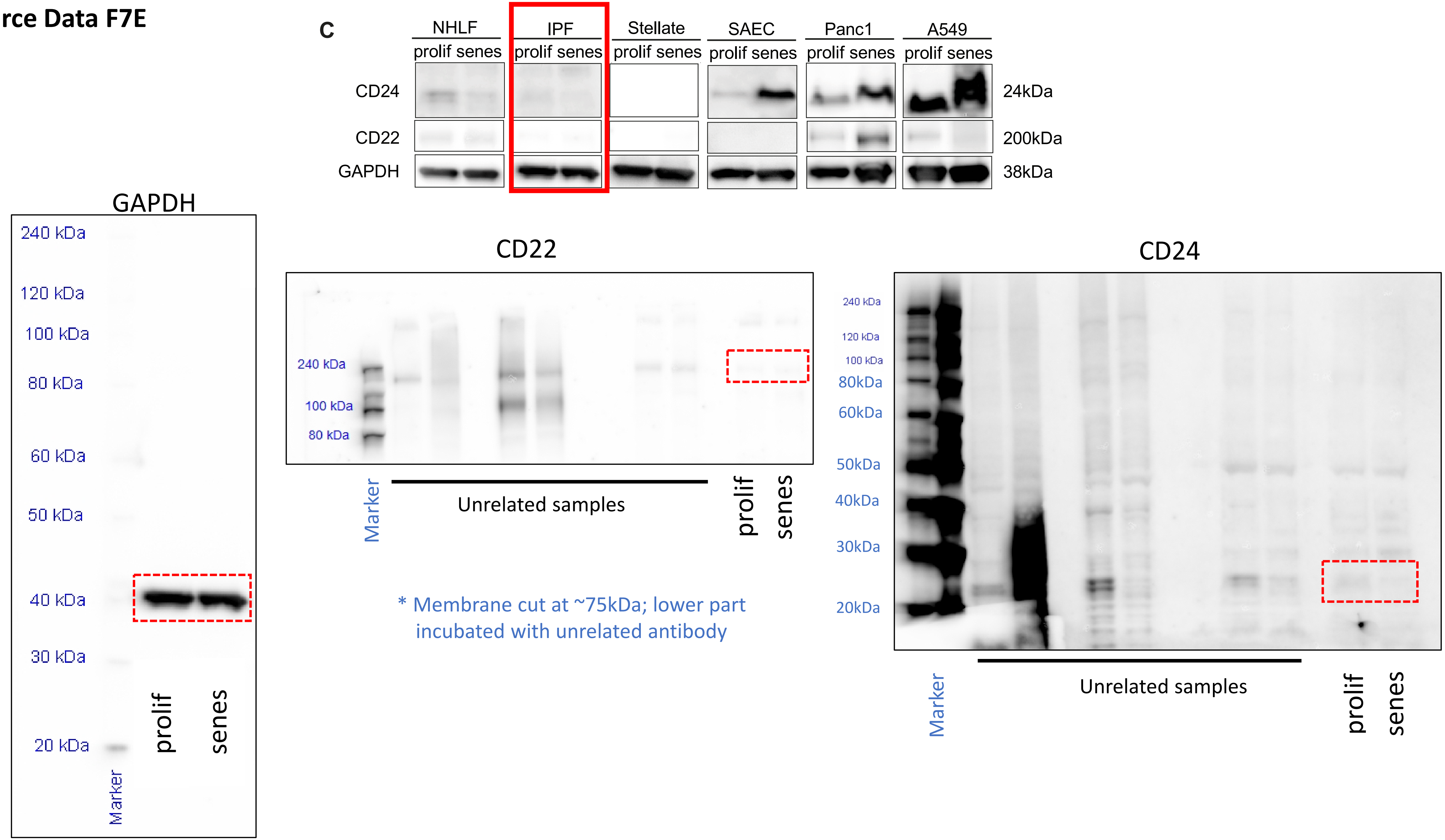

* Membrane cut at ~75kDa; lower part incubated with unrelated antibody

Source Data F7E

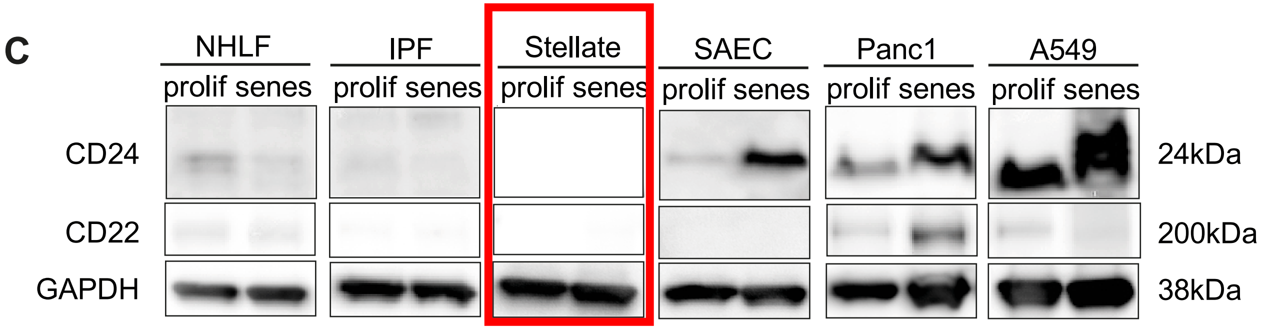

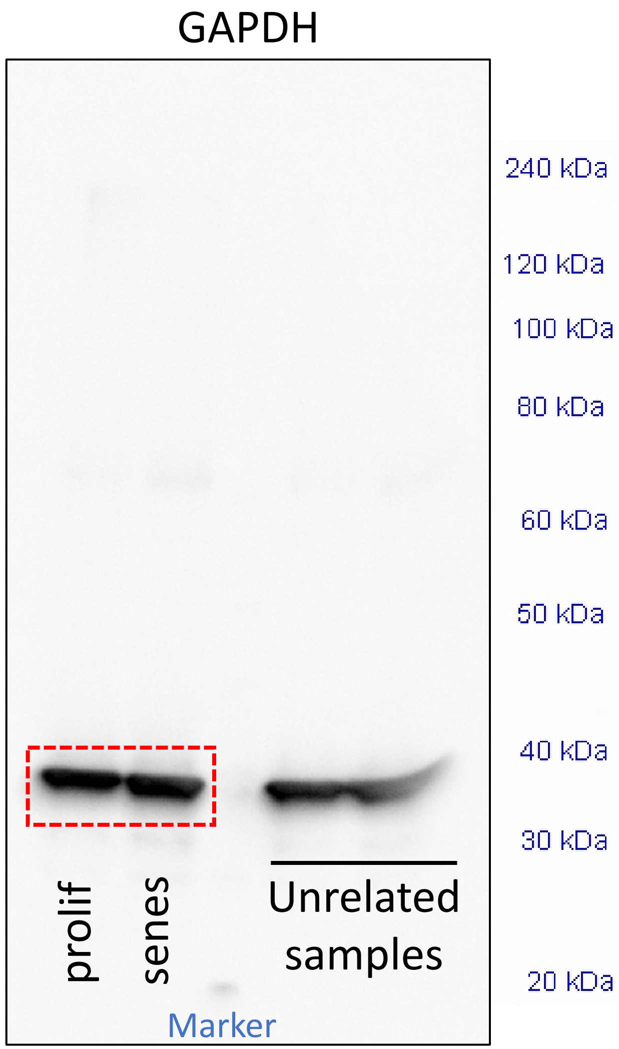

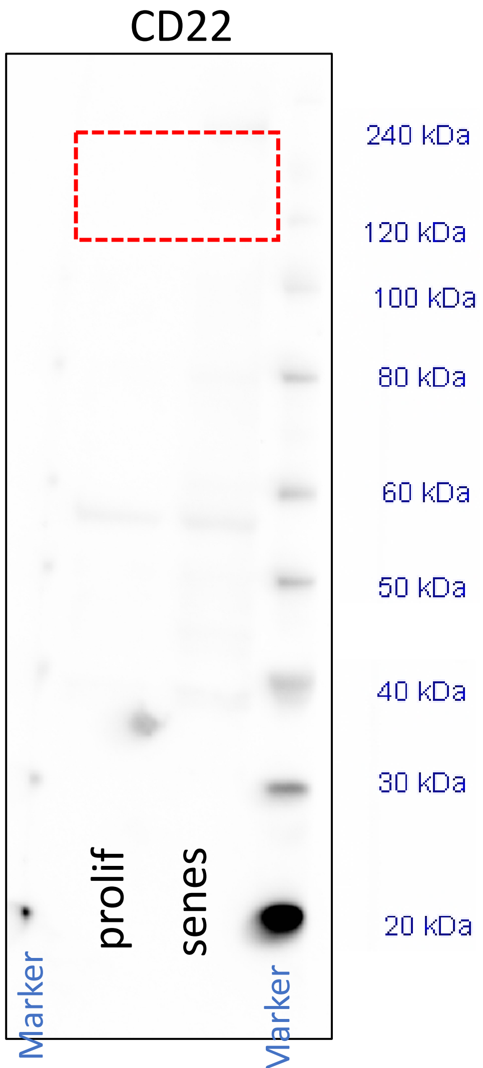

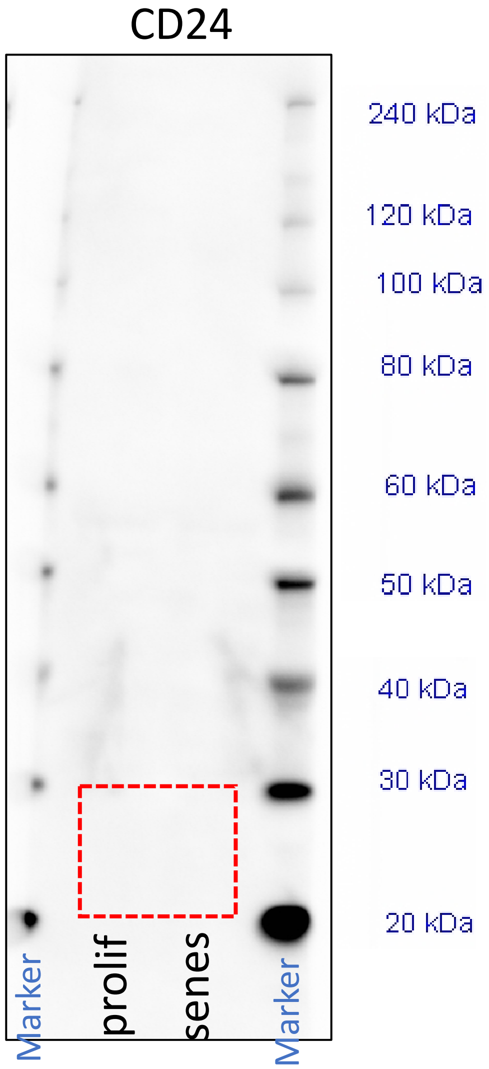

Source Data F7E

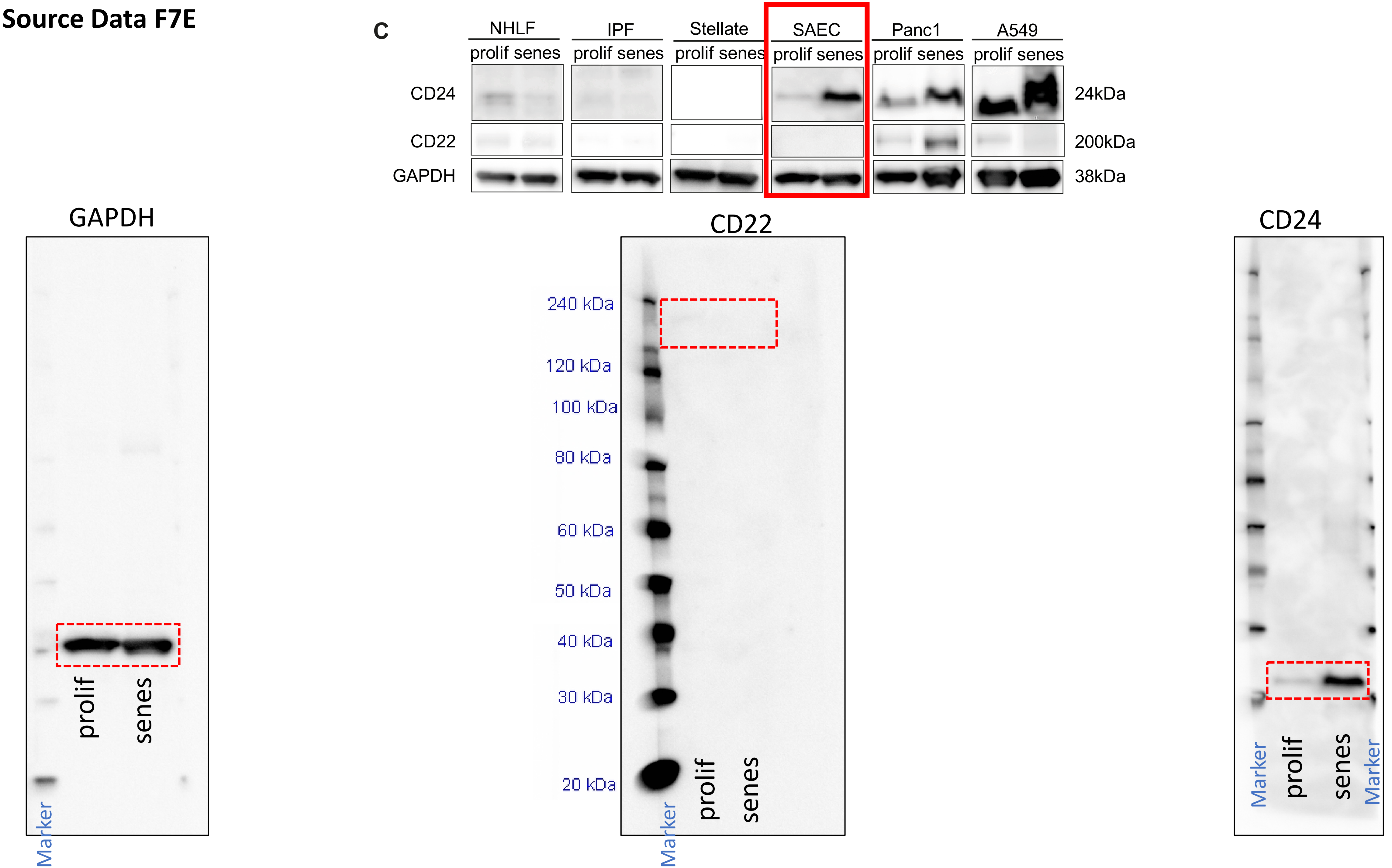

Source Data F7E

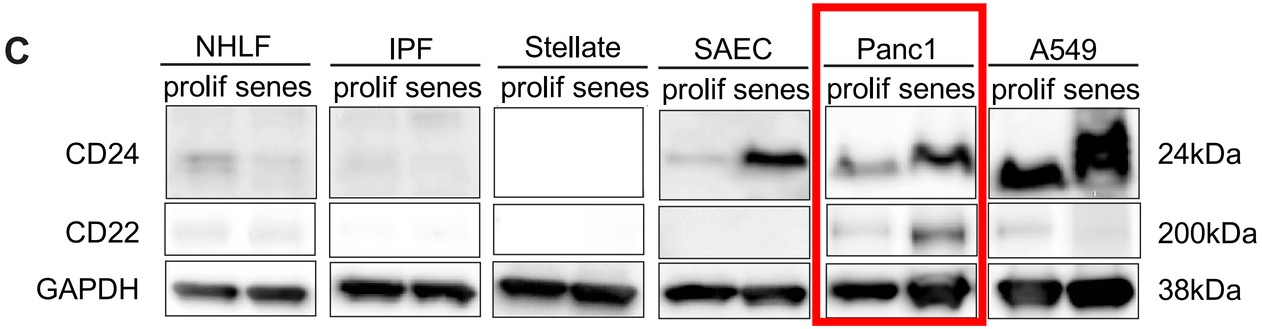

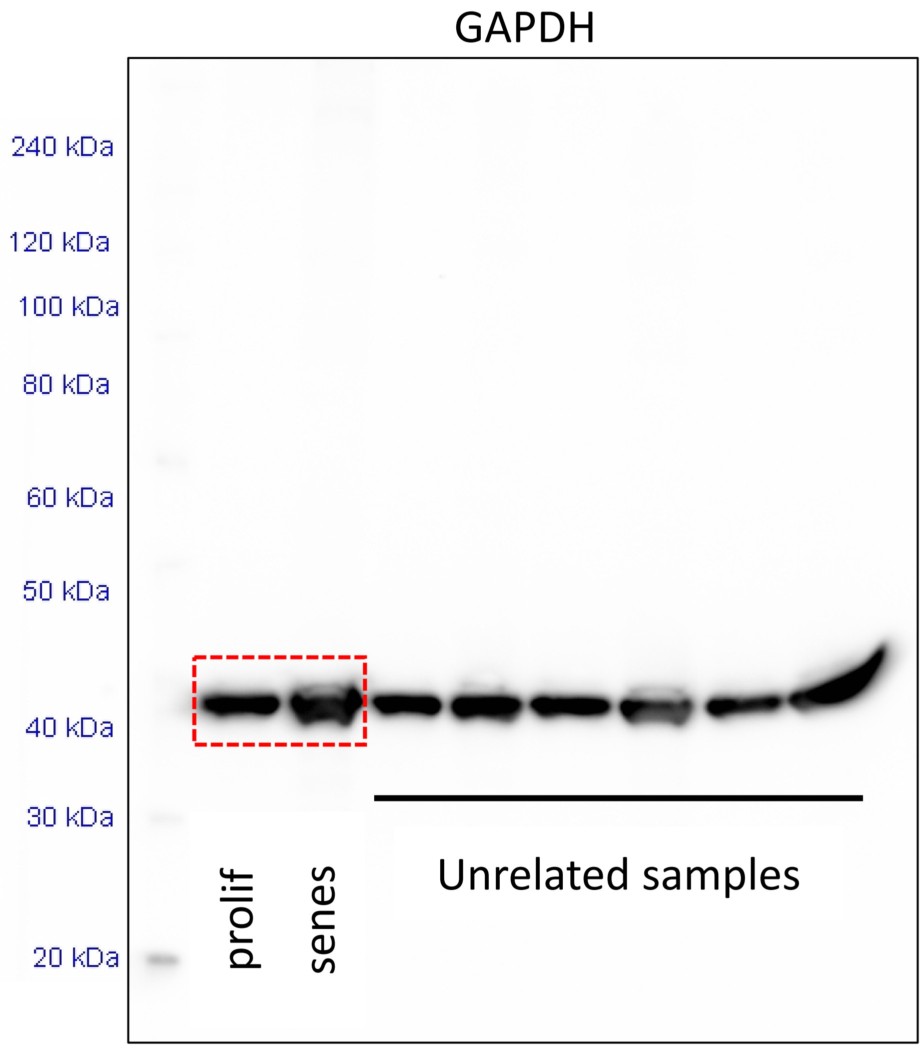

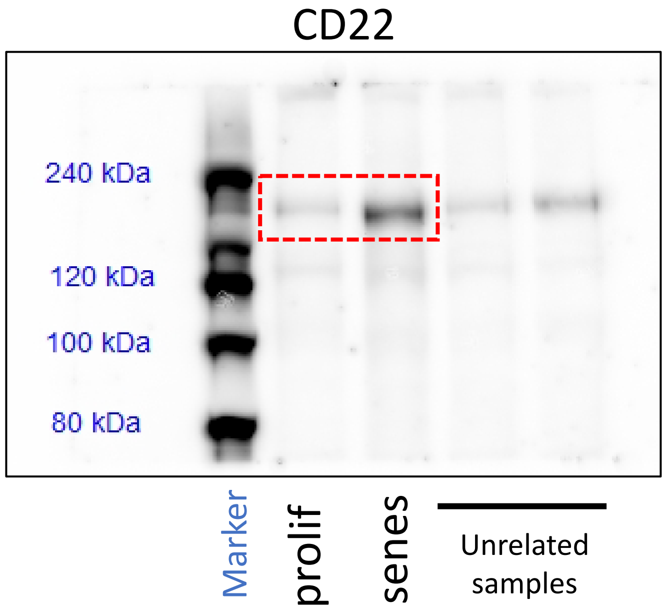

* Membrane cut at ~75kDa; lower part incubated with unrelated antibody

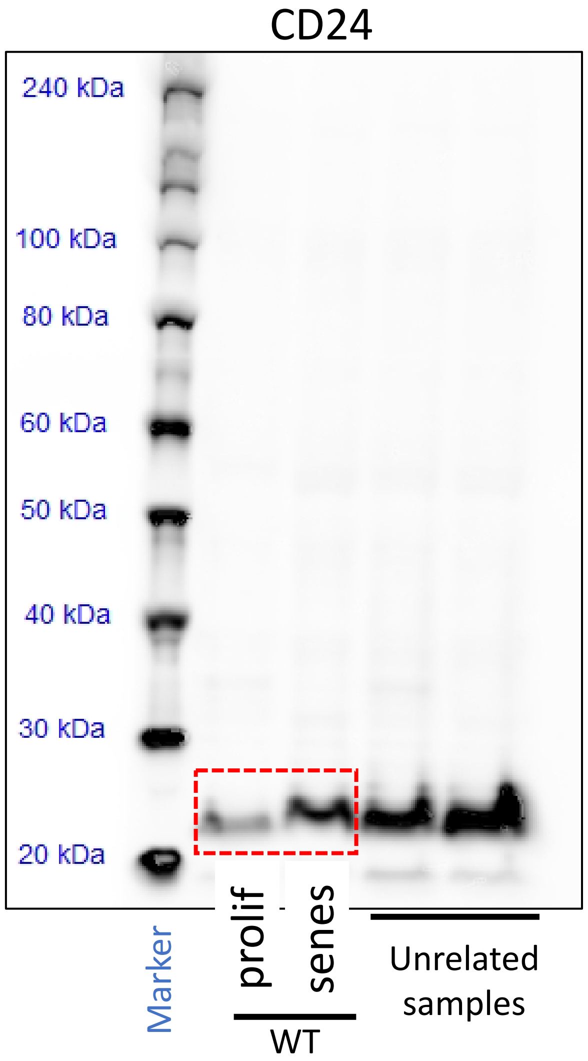

Source Data F7E

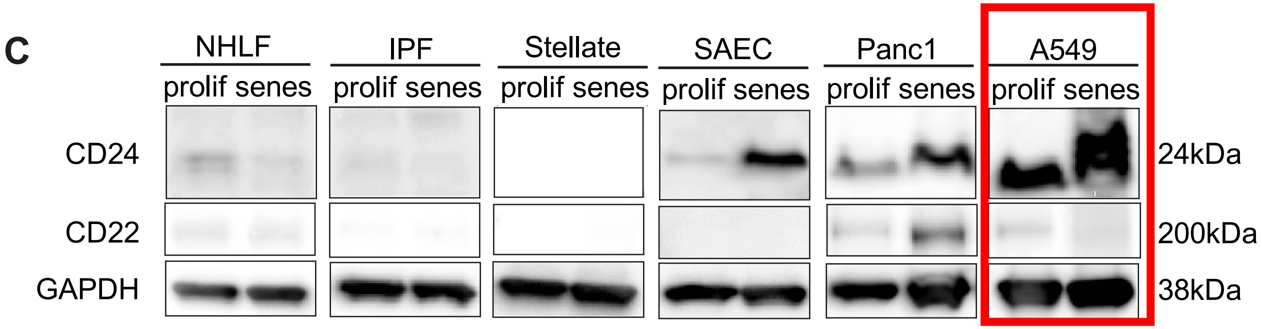

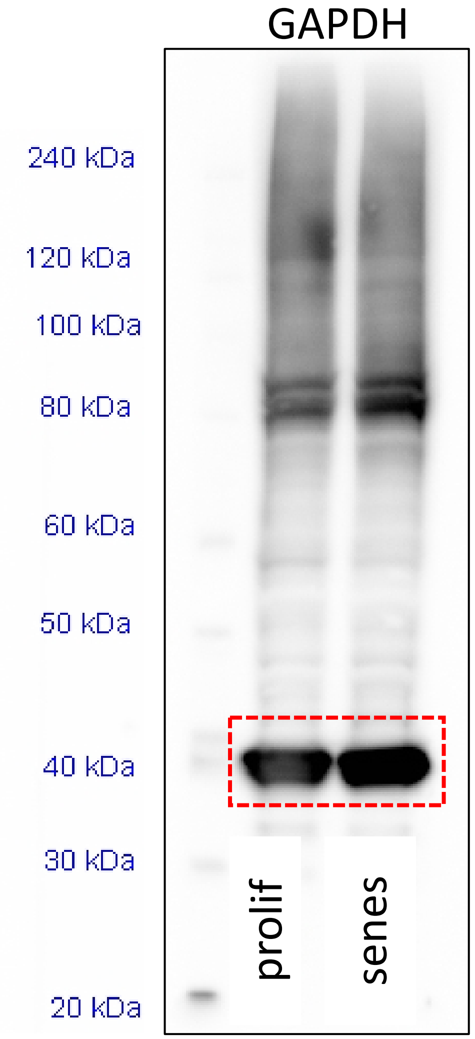

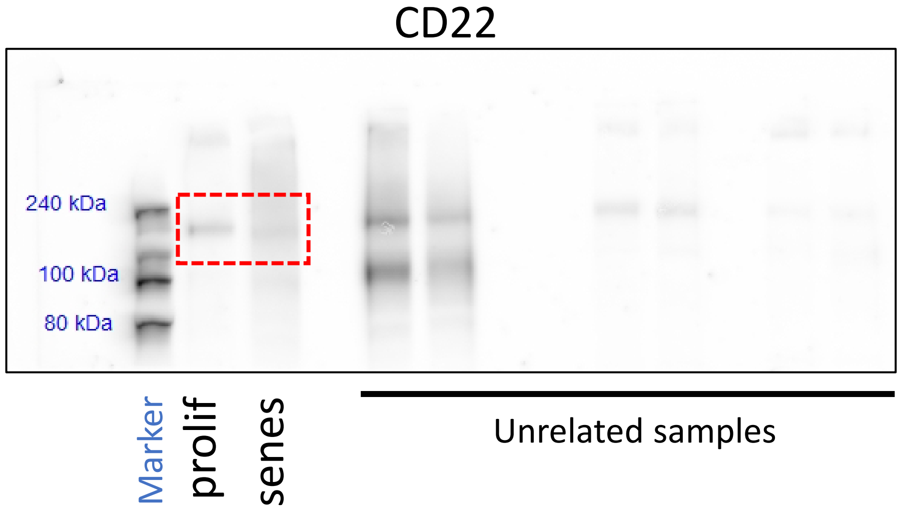

* Membrane cut at ~75kDa; lower part incubated with unrelated antibody

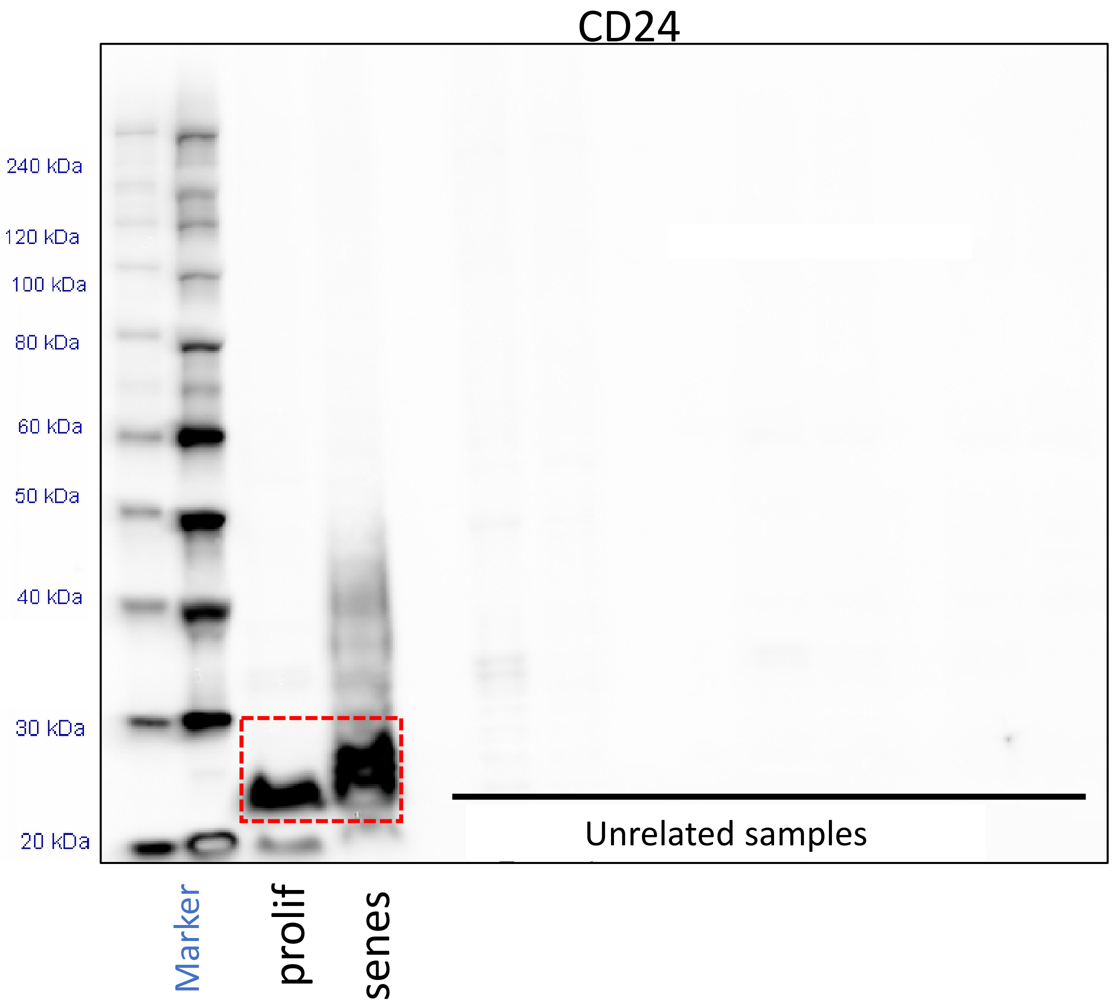

Supplement: SourceData FS3 — is the source file for Fig. S3. [file JCB_202207097_SourceDataFS3.pdf]
